# Long-Term Sequelae of COVID-19: A Systematic Review and Meta-Analysis of One-Year Follow-Up Studies on Post-COVID Symptoms

**DOI:** 10.3390/pathogens11020269

**Published:** 2022-02-19

**Authors:** Qing Han, Bang Zheng, Luke Daines, Aziz Sheikh

**Affiliations:** 1 Department of Social Policy and Intervention, University of Oxford, Oxford OX1 2ER, UK; qing.han@spi.ox.ac.uk; 2 Usher Institute, University of Edinburgh, Edinburgh EH16 4UX, UK; luke.daines@ed.ac.uk (L.D.); aziz.sheikh@ed.ac.uk (A.S.); 3 Department of Non-Communicable Disease Epidemiology, London School of Hygiene & Tropical Medicine, London WC1E 7HT, UK; 4 Ageing Epidemiology Research Unit, School of Public Health, Imperial College London, London W6 8RP, UK

**Keywords:** post-acute sequelae of COVID-19, long-COVID, prevalence, symptom, meta-analysis

## Abstract

Emerging evidence has shown that COVID-19 survivors could suffer from persistent symptoms. However, it remains unclear whether these symptoms persist over the longer term. This study aimed to systematically synthesise evidence on post-COVID symptoms persisting for at least 12 months. We searched PubMed and Embase for papers reporting at least one-year follow-up results of COVID-19 survivors published by 6 November 2021. Random-effects meta-analyses were conducted to estimate pooled prevalence of specific post-COVID symptoms. Eighteen papers that reported one-year follow-up data from 8591 COVID-19 survivors were included. Fatigue/weakness (28%, 95% CI: 18–39), dyspnoea (18%, 95% CI: 13–24), arthromyalgia (26%, 95% CI: 8–44), depression (23%, 95% CI: 12–34), anxiety (22%, 95% CI: 15–29), memory loss (19%, 95% CI: 7–31), concentration difficulties (18%, 95% CI: 2–35), and insomnia (12%, 95% CI: 7–17) were the most prevalent symptoms at one-year follow-up. Existing evidence suggested that female patients and those with more severe initial illness were more likely to suffer from the sequelae after one year. This study demonstrated that a sizeable proportion of COVID-19 survivors still experience residual symptoms involving various body systems one year later. There is an urgent need for elucidating the pathophysiologic mechanisms and developing and testing targeted interventions for long-COVID patients.

## 1. Introduction

Severe acute respiratory syndrome coronavirus 2 (SARS-CoV-2) is still rapidly spreading around the world, fuelled by the emergence of new variants. According to the World Health Organization (WHO) Coronavirus (COVID-19) Dashboard [1], there have been over 300 million confirmed cases of COVID-19 worldwide. A feature of COVID-19 that differs from other respiratory infections could be its multi-system symptomatology, complications, and long-term sequelae [2].

A growing body of evidence has shown that COVID-19 survivors may experience persistent symptoms affecting different organ systems after the acute phase of infection (also known as long-COVID [3]). Several previous systematic reviews and meta-analyses on long-COVID [2,4,5] estimated the prevalence of short- and medium-term persistent symptoms after COVID-19 infection, involving respiratory, cardiovascular, musculoskeletal, integumentary, gastrointestinal, endocrine, and neurological systems. A meta-analysis [4] of 33 papers investigating hospitalised and non-hospitalised COVID-19 survivors demonstrated that 63.2%, 71.9%, and 45.9% of patients experienced at least one persistent symptom at 30, 60, and ≥ 90 days after hospitalisation or onset, of which fatigue, dyspnoea, cough, anosmia, ageusia, and joint pain were the most prevalent symptoms. A meta-analysis [5] of 39 studies with up to seven months follow-up showed that the most frequently reported symptoms included weakness, fatigue, concentration impairment, and breathlessness. Another meta-analysis [2] of 15 papers reported over 50 persistent symptoms of COVID-19 experienced by COVID-19 survivors between 14–110 days post infection. 

However, no previous systematic review and meta-analysis has focused on longer-term persistent symptoms of COVID-19, and it remains unclear to what extent these broad classes of symptoms still persist after one year post infection. With the emerging data of longer-term follow-up of COVID-19 patients, it is important to investigate the natural history of COVID-19 symptomatology and whether the spectrum of long-lasting symptoms is different from that of the short- or medium-term manifestations previously described. 

We sought to systematically synthesise existing evidence on long-term post-COVID symptoms. We estimated the pooled prevalence and also summarised potential risk factors of post-COVID symptoms lasting for one year after infection. 

## 2. Materials and Methods

### 2.1. Search Strategy

The reporting of this study followed the Preferred Reporting Items for Systematic Reviews and Meta-analyses (PRISMA) reporting guideline [6]. We systematically searched PubMed and Embase databases for studies reporting one-year follow-up data for COVID-19 survivors published between 1 January 2021 and 6 November 2021, in English or Chinese. Studies were identified using search terms related to COVID-19, sequelae, and long-term follow-up. Medical Subject Headings (MeSH) terms and free text words were combined for the searches. Reference lists of relevant papers were checked for additional studies. Our detailed search strategies are presented in the Supplementary Methods. 

Two authors (Q.H. and B.Z.) independently screened the papers and performed data extraction and quality assessment. Any discrepancies were resolved following review of the corresponding papers by both authors and consensus discussion.

### 2.2. Inclusion/Exclusion Criteria

Studies were included if they used a cohort, cross-sectional, or case series design and reported data required for the estimation of pooled prevalence of post-COVID symptoms (i.e., sample size of COVID-19 survivors and the prevalence or proportion of specific symptoms). Eligible studies were required to have a mean/median follow-up time of at least 12 months. For studies with overlapping or identical participants, we included the study with the largest sample size. Studies were excluded from meta-analysis if they only recruited participants with reported residual symptoms or complications post COVID-19 or only recruited participants with a specific comorbidity (e.g., cancer patients). We also excluded studies with less than 50 COVID-19 survivors because of concerns regarding the precision and potential bias of prevalence estimates. Review papers without original data and conference abstracts without full text were excluded from this study.

### 2.3. Data Extraction

We extracted the following information from eligible studies: (1) sample size and prevalence or number of patients with specific post-COVID symptoms; (2) types and definitions of post-COVID symptoms; (3) follow-up period and follow-up method; and (4) population characteristics (country, age, sex, and severity of COVID-19 infection). We used the Cochrane recommended software PlotDigitizer (www.plotdigitizer.sourceforge.net/, accessed on 15 January 2022) to extract graphically presented prevalence data from two papers.

We also extracted results of risk factor analyses (if available) for a qualitatively synthesis of the risk factors for long-term post-COVID symptoms.

### 2.4. Study Quality Assessment

The study quality of eligible papers was evaluated using the “Joanna Briggs Institute (JBI) Critical Appraisal Checklist for Studies Reporting Prevalence Data” [7]. This checklist consists of nine items with four options (i.e., yes, no, unclear, not applicable). We assigned an overall quality rating of high, moderate, or low to each paper based on a qualitative evaluation of the nine items.

### 2.5. Statistical Analyses

Random-effects meta-analyses with inverse-variance method were conducted to estimate the pooled prevalence and its confidence interval (CI) of specific post-COVID symptoms in COVID-19 survivors [8]. The study-specific confidence intervals were estimated using the Wilson’s score method [8]. The meta-analyses were conducted only for symptoms assessed by three or more studies. The heterogeneity of prevalence estimates across studies was assessed by the I^2^ statistic and Q test in each analysis. For each symptom with > 10 studies, a funnel plot for prevalence estimates after logit transformation was created to graphically identify publication bias, followed by an Egger’s test of asymmetry.

To examine the robustness of the main findings, several sensitivity analyses were performed by: (1) excluding studies rated as poor quality and (2) repeating the meta-analyses using prevalence estimates after logit transformation or Freeman–Tukey double-arcsine transformation [8]. We also assessed the influence of each study on the overall estimate by recalculating the pooled prevalence after removing that study.

Statistical analyses were performed using Stata (version 14, StataCorp, College Station, TX, USA). All statistical tests were two-sided with the significance threshold of *p* < 0.05. 

## 3. Results

### 3.1. Search Results and Study Characteristics

**Figure 1:**
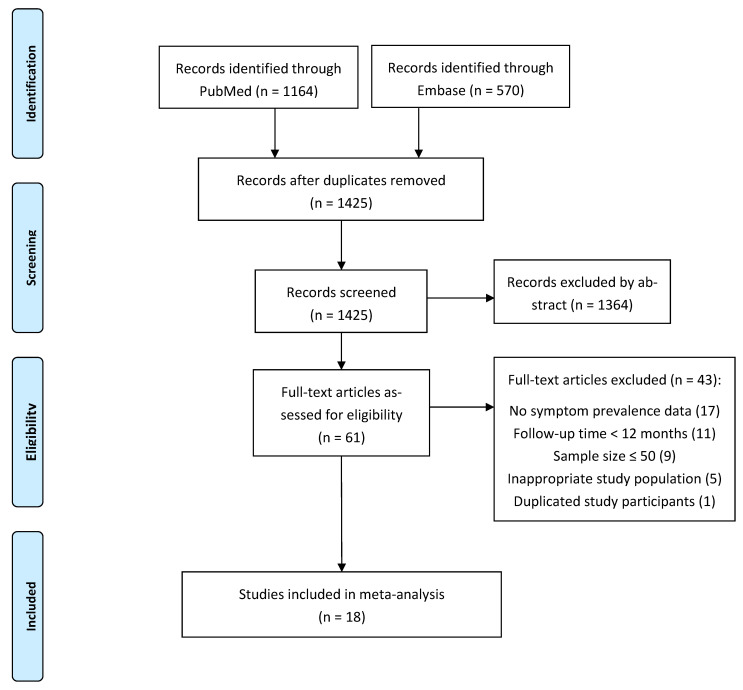
PRISMA flowchart.

The literature search in PubMed and Embase yielded 1425 records. After screening of abstract and full text, 18 eligible papers with a total of 8591 COVID-19 survivors were included in this systematic review and meta-analyses. Details of the literature screening process are displayed in the PRISMA flowchart (Figure 1). 

Characteristics of the 18 papers are summarised in Table 1. These one-year follow-up studies of COVID-19 patients were conducted in China (*n* = 7), Italy (*n* = 5), Spain (*n* = 4), and Germany (*n* = 2), with the longest follow-up time being a median of 401 days from the first SARS-CoV-2 positive swab [9]. All COVID-19 patients recruited by the 18 studies had confirmed SARS-CoV-2 infection. The sample size of the included studies varied from 51 to 2433 (median = 146). Thirteen studies recruited hospitalised COVID-19 patients, two studies with home-isolated COVID-19 patients with mild-to-moderate symptoms, and three studies with mixed samples of hospitalised and non-hospitalised patients. Thirteen studies only included adult patients, and the other five did not report the age range; no study focused on the long-term sequelae of children or adolescents with COVID-19. 

When assessing post-COVID symptoms, eight studies used clinical scales, including Acute Respiratory Tract Infection Questionnaire [10], five-item WHO Wellbeing Index questionnaire [11], modified Medical Research Council dyspnoea scale [12], Fatigue Severity Scale [13], Montreal Cognitive Assessment [14], Hospital Anxiety and Depression Scale (HADS) subscales [15], 14-item Hamilton Anxiety Rating Scale [16], 24-item Hamilton Depression Rating Scale [17], post-traumatic stress disorder (PTSD) checklist for DSM-5 [18], and Insomnia Severity Index [19]. The follow-up method included in-person visit (*n* = 11) and phone interview (*n* = 7).

**Table 1:** Study characteristics of 18 papers included in the meta-analyses.

First Author	Sample Size	Country	Mean/Median Age, Year	Only Adults	Male Proportion	Scale for Symptoms	Single-/Multi-Centre	Hospitalisation	Follow-Up Method	Follow-Up Period
Boscolo-Rizzo, P. [20]	304	Italy	47	Yes	0.39	Acute Respiratory Tract Infection Questionnaire	single centre	non-hospitalised	phone	12 months after symptom onset
Boscolo-Rizzo, P. [9]	100	Italy	49	Yes	0.61	5-item World Health Organization Wellbeing Index, Acute Respiratory Tract Infection Questionnaire	multi-centre	non-hospitalised	in-person visit	median of 401 days from the first SARS-CoV-2 positive swab
Catalán, I. P. [21]	76	Spain	64	Yes	0.62	-	single centre	hospitalised	phone	one year after hospital admission
Chai, C. [22]	432	China	65	-	0.49	-	multi-centre	hospitalised	in-person visit	median follow-up time from hospital admission was 12.2 (IQR 12.1-12.6) months
Fernández-de-las-Peñas, C. [23]	1950	Spain	61	-	0.53	-	multi-centre	hospitalised	phone	one year after hospital discharge
Gamberini, L. [24]	178	Italy	64	-	0.73	mMRC	multi-centre	ICU	in-person visit	one year after ICU discharge
Huang, L. [25]	1272	China	59	Yes	0.53	mMRC	single centre	hospitalised	in-person visit	12 months after symptom onset
Latronico, N. [26]	51	Italy	60	Yes	0.77	Fatigue Severity Score, Montreal Cognitive Assessment, Hospital Anxiety and Depression Scale subscales, PTSD checklist for DSM-5, Insomnia Severity Index	single centre	ICU	in-person visit	12 months after ICU discharge
Liu, T. [27]	486	China	63	-	0.46	-	single centre	hospitalised	in-person visit	12 months after discharge
Maestre-Muñiz, M. M. [28]	543	Spain	65	Yes	-	-	single centre	mixed	phone	12 months after discharge
Maestrini, V. [29]	118	Italy	71	-	0.57	-	single centre	hospitalised	phone	mean of 347 ± 10 days from COVID-19 diagnosis
Méndez, R. [30]	171	Spain	58	Yes	0.58	a battery of standardised instruments for the cognitive functioning; subjective cognitive complaints; anxiety, depression, and PTSD	single centre	hospitalised	phone	12 (±1) months after hospital discharge
Rank, A. [31]	83	Germany	42	Yes	0.76	-	single centre	mixed	in-person visit	12 months after onset of COVID-19
Seeßle, J. [32]	96	Germany	57	Yes	0.45	-	single centre	mixed	in-person visit	12 months after symptom onset
Wu, X. [33]	83	China	60	Yes	0.57	mMRC	single centre	hospitalised	in-person visit	12 months after discharge
Zhan, Y. [34]	121	China	49	Yes	0.41	-	single centre	hospitalised	in-person visit	one year after diagnosis
Zhang, X. [35]	2433	China	60	Yes	0.50	-	multi-centre	hospitalised	phone	median (IQR) time from discharge to follow-up was 364 (357–371) days
Zhao, Y. [36]	94	China	48	Yes	0.57	14-item Hamilton Anxiety Rating Scale, 24-item Hamilton Depression Rating Scale, mMRC	multi-centre	hospitalised	in-person visit	median duration from symptom onset to follow-up visit was 366 (355, 376) days; median time from hospital discharge to follow-up visit was 345 (333, 349) days.

Note: “-“ refers to information unclear or not applicable. IQR, interquartile range; ICU, intensive care unit; mMRC, modified Medical Research Council dyspnoea scale; PTSD, post-traumatic stress disorder; DSM-5, Diagnostic and Statistical Manual of Mental Disorders, 5th Edition.

### 3.2. Assessment of Risk of Bias

The study quality of eight papers was rated as high, while another eight papers were rated moderate quality, and two with low quality (Supplementary Table S1). Sources of potential bias among these studies were lack of representativeness of the study sample (12 studies were single-centre studies), small sample size, low response rate, lack of validated scales for symptom assessment, and lack of standard and reliable follow-up methods (especially for phone interviews). 

### 3.3. Prevalence of One-Year Post-COVID Symptoms among COVID-19 Survivors

**Figure 2:**
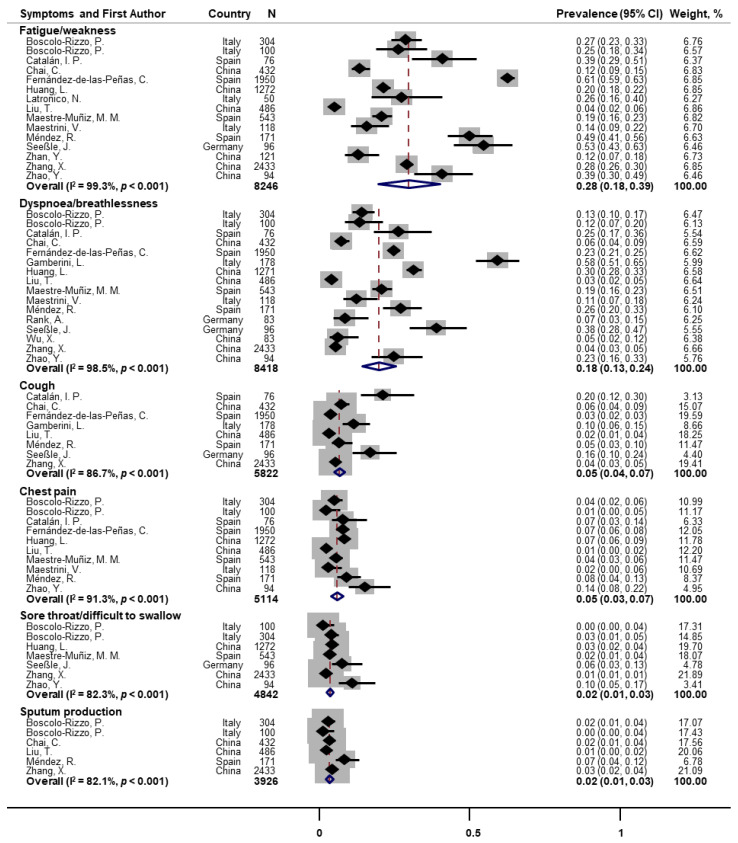
Forest plot for prevalence of post-COVID fatigue and respiratory symptoms. Note: There were missing values for dyspnoea and fatigue in papers of Huang et al. [25] and Latronico et al. [26].

Random-effects meta-analyses were conducted for 27 individual symptoms separately. Fifteen and sixteen papers assessed fatigue/weakness and dyspnoea/breathlessness at one-year follow-up, the pooled prevalence of which were 28% (95% CI: 18–39) and 18% (95% CI: 13–24), respectively (Figure 2). Substantial heterogeneity across studies was observed (I^2^ = 99.3% and 98.5%, both *p* < 0.001). Other post-COVID respiratory symptoms were also reported but with much lower prevalence. The pooled prevalence was 5% for cough (*n* = 8; 95% CI: 4–7; I^2^ = 86.7%), 5% for chest pain (*n* = 10; 95% CI: 3–7; I^2^ = 91.3%), 2% for sore throat/difficulty swallowing (*n* = 7; 95% CI: 1–3; I^2^ = 82.3%), and 2% for sputum production (*n* = 6; 95% CI: 1–3; I^2^ = 82.1%; Figure 2). 

**Figure 3:**
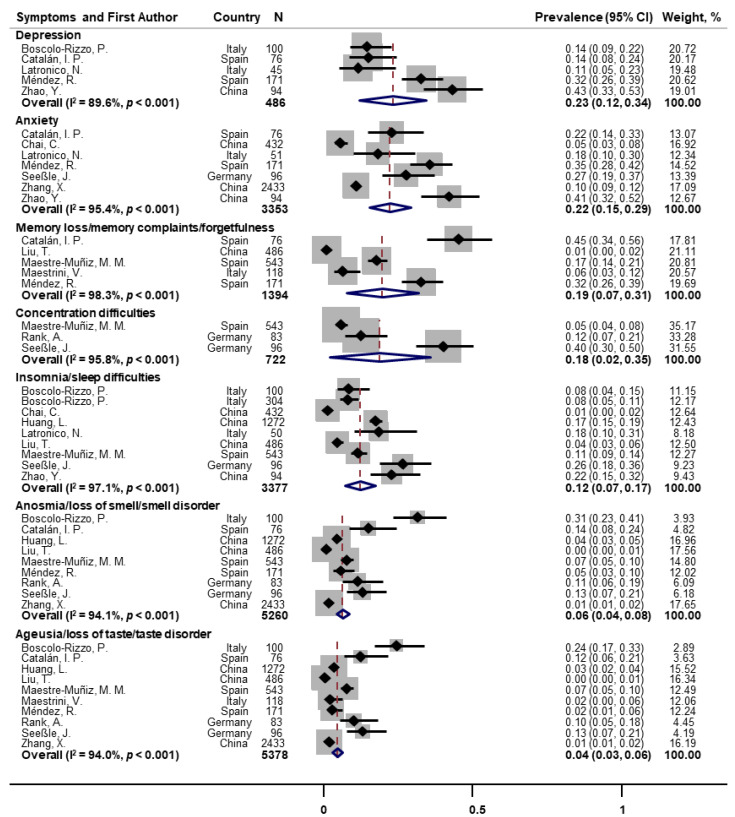
Forest plot for prevalence of post-COVID mental health and cognitive symptoms. Note: There were missing values for depression and insomnia in the paper of Latronico et al. [26].

Mental health and cognitive symptoms were frequently reported among COVID-19 survivors (Figure 3). The pooled prevalence for depression and anxiety symptoms were 23% (*n* = 5; 95% CI: 12–34; I^2^ = 89.6%) and 22% (*n* = 7; 95% CI: 15–29; I^2^ = 95.4%), respectively. Memory loss/memory complaints/forgetfulness and concentration difficulties were also commonly experienced, with the pooled prevalence of 19% (*n* = 5; 95% CI: 7–31; I^2^ = 98.3%) and 18% (*n* = 3; 95% CI: 2–35; I^2^ = 95.8%), respectively. Insomnia/sleep difficulties had a pooled prevalence of 12% (*n* = 9; 95% CI: 7–17; I^2^ = 97.1%). In addition, the pooled prevalence of anosmia/loss of smell/smell disorder and ageusia/loss of taste/taste disorder were 6% (*n* = 9; 95% CI: 4–8; I^2^ = 94.1%) and 4% (*n* = 10; 95% CI: 3–6; I^2^ = 94.0%), respectively.

**Figure 4:**
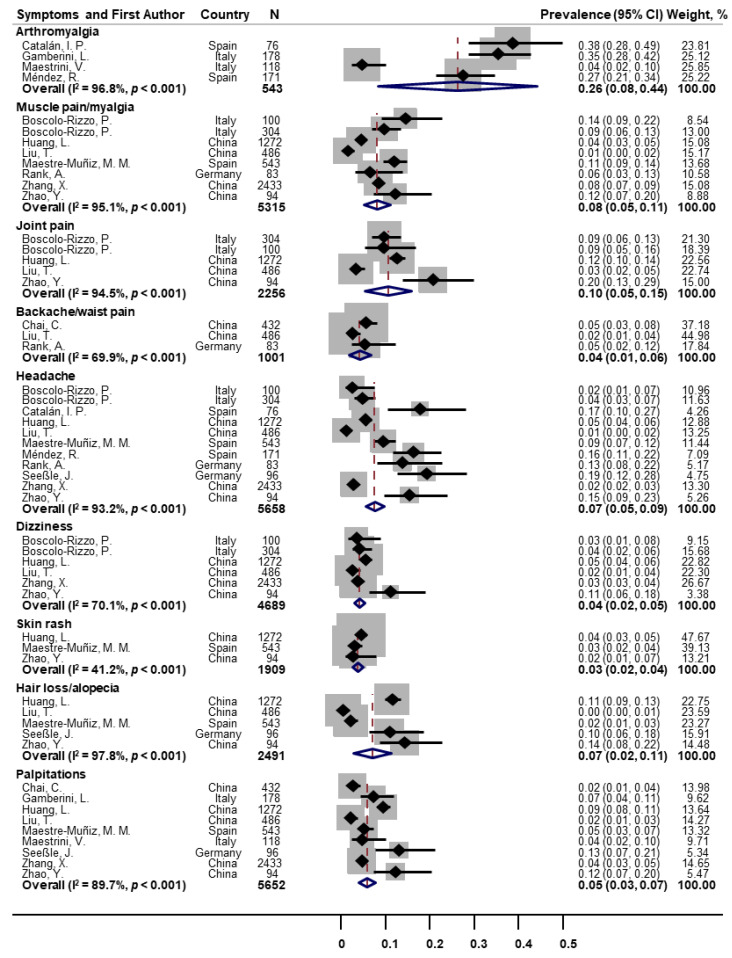
Forest plot for prevalence of other post-COVID symptoms.

As for other classes of symptoms (Figure 4), the pooled prevalence was 26% for arthromyalgia (*n* = 4; 95% CI: 8–44; I^2^ = 96.8%), 8% for muscle pain/myalgia (*n* = 8; 95% CI: 5–11; I^2^ = 95.1%), 10% for joint pain (*n* = 5; 95% CI: 5–15; I^2^ = 94.5%), 4% for backache/waist pain (*n* = 3; 95% CI: 1–6; I^2^ = 69.9%), 7% for headache (*n* = 11; 95% CI: 5–9; I^2^ = 93.2%), 4% for dizziness (*n* = 6; 95% CI: 2–5; I^2^ = 70.1%), 3% for skin rash (*n* = 3; 95% CI: 2–4; I^2^ = 41.2%), 7% for hair loss/alopecia (*n* = 5; 95% CI: 2–11; I^2^ = 97.8%), and 5% for palpitations (*n* = 9; 95% CI: 3–7; I^2^ = 89.7%). 

Fever, vomiting/nausea, diarrhoea, abdominal pain, and loss of appetite were uncommon symptoms at one-year follow-up, with the pooled prevalence of 0.3% (*n* = 7; 95% CI: 0.0–1.2; I^2^ = 79.8%), 0.7% (*n* = 5; 95% CI: 0.0–2.2; I^2^ = 83.5%), 1.3% (*n* = 6; 95% CI: 0.2–2.8; I^2^ = 80.7%), 0.4% (*n* = 3; 95% CI: 0.0–1.8; I^2^ = 70.8%), and 1.0% (*n* = 4; 95% CI: 0.0–3.4; I^2^ = 90.8%), respectively (Supplementary Table S2). Other symptoms, such as hearing loss, PTSD symptom, rhinorrhoea/runny nose, and chest tightness, were only reported by one or two studies with one-year follow-up, the details of which are displayed in Supplementary Table S2.

The funnel plots (Supplementary Figures S1–S3) and Egger’s tests for meta-analyses of fatigue/weakness, dyspnoea/breathlessness, and headache (where *n* > 10) showed no evidence of publication bias (*p* > 0.10). Results of the sensitivity analyses were consistent with the main findings (Supplementary Table S3). The influential analysis indicated no single study had a major impact on the pooled prevalence estimate (Supplementary Table S3). 

### 3.4. Evidence of Risk Factors for One-Year Post-COVID Symptoms

Of the 18 included studies, 10 examined potential risk factors for post-COVID symptoms at one-year follow-up. Three studies [25,32,35] found that female patients had significantly higher risk of experiencing symptoms one-year post-COVID than males, and one study [20] detected a borderline statistical significance. Inconsistent results were reported for age, with two studies [25,35] showing a positive association with post-COVID symptoms, one showing a negative association [32], one showing a higher risk in the middle age group (40–54 years old) [20], and one reporting no association [36]. One study [20] found a positive association between body mass index (BMI) and risk of long-term symptoms. 

Three studies [25,34,35] showed a higher risk of long-term symptoms in severe/critical patients than non-severe patients in terms of the acute infection status, one study [36] found such association only for muscle fatigue but not for other symptoms, and another study [32] did not detect the association. In addition, one study [28] reported higher prevalence of long-term symptoms in hospitalised patients than non-hospitalised patients, and another study [24] found a positive association between duration of invasive mechanical ventilation and post-COVID dyspnoea at one-year follow-up.

A large-scale cohort study in China [25] found no association of education level, smoking status, and comorbidity with one-year post-COVID symptoms. Another large-scale study in Spain [23] investigating persistent cough (which was reported by 2.5% of recruited COVID-19 survivors) showed no association between cough and sex, age, BMI, initial disease severity, days at hospital, and smoking status. One study [21] found that corticosteroids therapy received during hospitalisation was associated with lower risk of headache, dysphagia, chest pain, and depression at one-year follow-up; however, the afore-mentioned Chinese cohort study [25] showed that corticosteroids therapy was associated with increased risk of fatigue or muscle weakness and had no association with anxiety or depression one year later.

## 4. Discussion

We conducted a comprehensive evidence synthesis of the prevalence and risk factors for post-COVID symptoms lasting for one year after acute infection. The meta-analyses on prevalence data showed that common residual symptoms among COVID-19 survivors at one-year post infection included fatigue/weakness (28%), dyspnoea (18%), arthromyalgia (26%), depression (23%), anxiety (22%), memory loss (19%), concentration difficulties (18%), and insomnia (12%). The qualitative review of evidence on risk factors suggested that females [20,25,32,35] and those with severe/critical COVID-19 infection [25,34,35] were at higher risk of experiencing long-term post-COVID symptoms.

Reliable estimates for the prevalence of long-COVID symptoms among COVID-19 survivors are essential for policy makers and clinicians to anticipate associated healthcare burden and inform decisions on the allocation of healthcare resources. To be noted, some major COVID-19 symptoms at the acute phase of infection, such as cough, fever, and sore throat [37], were not frequently experienced one year later. Although loss of taste or smell was a common symptom at the acute phase [37] and also during short- and medium-term follow-up [4], it seemed to improve over time. In addition, consistent with a previous meta-analysis on prolonged gastrointestinal symptoms in COVID-19 survivors [38], we found a low prevalence of nausea, vomiting, diarrhoea, abdominal pain, and loss of appetite at one-year follow-up.

Several studies included in this meta-analysis examined the evolution of post-COVID symptoms by comparing data from multiple follow-up visits. A large-scale cohort study of hospitalised COVID-19 patients in Wuhan, China [25], showed that the percentage of having at least one persisting symptom significantly decreased from 68% at 6 months to 49% at 12 months post COVID, but mixed results were found for the trajectory of individual symptoms. Another Chinese cohort study [27] also found that the percentage of having at least one symptom decreased from 51.2% at 3 months post-discharge to 40.0% and 28.4% at 6-month and 12-month visits. However, a small-scale cohort study in Germany [32] showed that between 5 months and 12 months post COVID, the prevalence of hair loss decreased significantly from 26.1% to 10.4%, but the prevalence of fatigue and dyspnoea increased (from 41.7% to 53.1% and from 27.1% to 37.5%, respectively). Another small-scale Italian cohort study of survivors after intensive care unit discharge [26] found that cognitive impairment significantly improved (from 28% at 3 months to 16% at 12 months), but no significant changes were observed for severe fatigue, depression, anxiety, insomnia, and PTSD symptoms.

One important methodological concern when interpreting these studies on post-COVID symptoms is how to distinguish post-COVID symptoms from pre-COVID symptoms or the population’s baseline level, especially for symptoms with a relatively low prevalence. In fact, six of the included studies [21,23,28,32,35,36] asked participants to take into account their pre-COVID state when reporting post-COVID symptoms (i.e., new or worsening symptoms compared with pre-COVID baseline) though such measurement relied on participants’ recall accuracy. The afore-mentioned large-scale cohort study in Wuhan, China [25], included a matched non-COVID-19 control group, which showed that COVID-19 survivors had a significantly higher prevalence of all individual symptoms assessed at the one-year follow-up visit than the control population (proportion of having any one of the symptoms: 66% vs. 33%, *p* < 0.001).

The current body of evidence suggested that female sex [20,25,32,35] and severe/critical acute infection [25,34,35] could be risk factors for experiencing long-term post-COVID symptoms. A cohort study with 342 COVID-19 patients [39] found that time to complete recovery (no ongoing symptoms) was significantly longer in those with moderate and severe/critical initial illness than mild cases, and at least one persistent symptom was reported by 16.4%, 49.5%, and 52.5% of participants in mild, moderate, and severe/critical groups at one year after infection, respectively. However, the pathophysiologic mechanisms of those long-lasting post-COVID symptoms remained unclear. 

Several limitations of this study should be noted. This systematic review and meta-analysis focused on subjective symptoms of COVID-19 patients. Future systematic evidence syntheses on other types of potential long-term sequelae, such as persistent pulmonary function impairment, lung imaging abnormalities, limited exercise capacity, systemic inflammation, and incident clinical complications, are needed. In addition, caution is needed in interpreting pooled estimates of symptom prevalence (especially those with wide confidence intervals) due to the heterogeneity across studies. Little evidence was available for long-term follow-up of children or adolescents with COVID-19, making future research into the evolution of symptoms for this population warranted. Finally, it is likely that the one-year follow-up studies included in this meta-analysis covered the initial waves of the pandemic; thus, our findings mainly applied to those who experienced infection in the Wild and Alpha eras. Future research on infection by the Delta or Omicron variant and its long-term consequences could help elucidate how the COVID-19 pandemic evolves over time.

In conclusion, this systematic review and meta-analysis demonstrated that multiple physical, cognitive, and mental health symptoms persist for at least one year in a sizeable proportion of COVID-19 survivors. Persistent symptoms could be more evident in females and those with more severe acute COVID-19 infection. There is an urgent need to elucidate the underlying pathophysiologic mechanisms and for trials of interventions to treat or prevent the persistence of these long-lasting symptoms. 

## Supplementary Materials

The following supporting information can be downloaded at: https://www.mdpi.com/article/10.3390/pathogens11020269/s1, Supplementary methods; Figure S1: Funnel plot of meta-analysis for prevalence of post-COVID fatigue/weakness; Figure S2: Funnel plot of meta-analysis for prevalence of post-COVID dyspnoea/breathlessness; Figure S3: Funnel plot of meta-analysis for prevalence of post-COVID headache; Table S1: Risk of bias assessments of 18 eligible papers; Table S2: Information on other reported post-COVID symptoms; Table S3: Sensitivity analyses for prevalence of post-COVID symptoms.
