# Supplementary material for: Long-Term Sequelae of COVID-19: A Systematic Review and Meta-Analysis of One-Year Follow-Up Studies on Post-COVID Symptoms
Source: Pathogens. 2022 Feb 19;11(2):269. doi: 10.3390/pathogens11020269 (PMC8875269; doi:10.3390/pathogens11020269)
Supplement: Supplementary file 1 [file pathogens-11-00269-s001.zip › pathogens-1578532-supplementary.pdf]

Supplementary materials

Supplementary Methods. Search strategies.

Supplementary Table S1. Risk of bias assessments of 18 eligible papers.

Supplementary Table S2. Information on other reported post-COVID symptoms.

Supplementary Table S3. Sensitivity analyses for prevalence of post-COVID symptoms.

Supplementary Figure S1. Funnel plot of meta-analysis for prevalence of post-COVID fatigue/weakness.

Supplementary Figure S2. Funnel plot of meta-analysis for prevalence of post-COVID dyspnoea/breathlessness.

Supplementary Figure S3. Funnel plot of meta-analysis for prevalence of post-COVID headache.

Supplementary Methods. Search strategies.

PubMed:

#1: COVID-19 OR "COVID 19" [MeSH Terms] OR COVID-2019 OR SARS-CoV-2 OR 2019-nCoV OR 2019-SARS-CoV-2

#2: survivor* OR recover* OR persistent OR follow up OR discharge* OR sequela* OR long Covid

#3: ("long term" AND follow up) OR 1 year OR one year OR 12 months OR twelve months

#4: #1 AND #2 AND #3 Filters: Humans, English, Chinese, from 2021 – 2021

Embase:

1	exp coronavirus disease 2019/
2	(survivor* or recover* or persistent or follow up or discharge* or sequela* or long Covid).tw.
3	(("long term" and follow up) or 1 year or one year or 12 months or twelve months).tw.
4	1 and 2 and 3
5	limit 4 to (human and (chinese or english) and yr="2021")
6	limit 5 to conference abstract
7	5 not 6

Supplementary Table S1. Risk of bias assessments of 18 eligible papers.

**First author**	**1. Sample frame appropriate to address the target population?**	**2. Were study participants sampled in an appropriate way?**	**3. Was the sample size adequate?**	**4. Were the study subjects and the setting described in detail?**	**5. Was the data analysis conducted with sufficient coverage of the identified sample?**	**6. Were valid methods used for the identification of the condition?**	**7. Was the condition measured in a standard, reliable way for all participants?**	**8. Was there appropriate statistical analysis?**	**9. Was the response rate adequate, and if not, was the low response rate managed appropriately?**	**Overall quality rating**
Boscolo-Rizzo, P.	No	Yes	Yes	No	Unclear	Yes	No	Yes	Yes	High
Boscolo-Rizzo, P.	Yes	Yes	No	Yes	Unclear	Yes	Yes	Yes	Unclear	High
Catalán, I. P.	No	Yes	No	Yes	Unclear	No	No	Yes	Yes	Moderate
Chai, C.	No	Yes	Yes	Yes	Unclear	No	Yes	Yes	Yes	High
Fernández-de-las-Peñas, C.	No	Yes	Yes	Yes	Unclear	No	No	Yes	Yes	Moderate
Gamberini, L.	Yes	Yes	No	Yes	Unclear	Yes	Yes	Yes	Yes	High
Huang, L.	No	Yes	Yes	Yes	Unclear	Yes	Yes	Yes	Yes	High
Latronico, N.	No	Yes	No	Yes	Unclear	Yes	Yes	Yes	No	Moderate
Liu, T.	No	Yes	Yes	Yes	Unclear	No	Yes	Yes	No	Moderate

Maestre-Muñiz, M. M.	No	Yes	Yes	Yes	Unclear	Yes	No	Yes	Yes	High
Maestrini, V.	No	Yes	No	Yes	Unclear	No	No	Yes	Yes	Low
Méndez, R.	No	Yes	No	No	Unclear	Yes	No	No	Unclear	Low
Rank, A.	No	Yes	No	Yes	Unclear	No	Yes	Yes	Yes	Moderate
Seeßle, J.	No	Yes	No	Yes	Unclear	No	Yes	Yes	Unclear	Moderate
Wu, X.	No	Yes	No	Yes	Unclear	Yes	Yes	Yes	Yes	High
Zhan, Y.	No	Yes	No	Yes	Unclear	No	Yes	Yes	Yes	Moderate
Zhang, X.	No	Yes	Yes	Yes	Unclear	No	No	Yes	Yes	High
Zhao, Y.	Yes	Yes	No	Yes	Unclear	Yes	Yes	Yes	No	Moderate

Note: The risk of bias assessment was conducted based on the "Joanna Briggs Institute (JBI) Critical Appraisal Checklist for Studies Reporting Prevalence Data" (https://jbi.global/critical-appraisal-tools).

Supplementary Table S2. Information on other reported post-COVID symptoms.

Symptoms	First author	Sample size	Country	Prevalence of symptom
Fever	Boscolo-Rizzo, P.	304	Italy	0.015
	Boscolo-Rizzo, P.	100	Italy	0
	Catalán, I. P.	76	Spain	0
	Maestre-Muñiz, M. M.	543	Spain	0
	Méndez, R.	171	Spain	0.012
	Seeßle, J.	96	Germany	0.016
	Zhang, X.	2433	China	0
Vomiting/nausea	Boscolo-Rizzo, P.	304	Italy	0.021
	Boscolo-Rizzo, P.	100	Italy	0.01
	Liu, T.	486	China	0
	Seeßle, J.	96	Germany	0.044
	Zhang, X.	2433	China	0.002
Diarrhoea	Boscolo-Rizzo, P.	304	Italy	0.019
	Boscolo-Rizzo, P.	100	Italy	0.02
	Catalán, I. P.	76	Spain	0.053
	Liu, T.	486	China	0
	Seeßle, J.	96	Germany	0.033
	Zhang, X.	2433	China	0.007
Abdominal pain	Boscolo-Rizzo, P.	304	Italy	0.01
	Boscolo-Rizzo, P.	100	Italy	0.01
	Liu, T.	486	China	0
Loss of appetite	Boscolo-Rizzo, P.	304	Italy	0.012
	Boscolo-Rizzo, P.	100	Italy	0.01

Symptoms	First author	Sample size	Country	Prevalence of symptom
	Huang, L.	1272	China	0.03
	Liu, T.	486	China	0
Problems breathing	Boscolo-Rizzo, P.	304	Italy	0.067
	Boscolo-Rizzo, P.	100	Italy	0.03
Dry cough	Boscolo-Rizzo, P.	304	Italy	0.043
	Boscolo-Rizzo, P.	100	Italy	0.02
Chest congestion	Chai, C.	432	China	0.09
Chest tightness	Liu, T.	486	China	0.033
	Zhang, X.	2433	China	0.13
Rhinorrhoea/runny nose	Catalán, I. P.	76	Spain	0.197
Blocked nose	Boscolo-Rizzo, P.	304	Italy	0.036
	Boscolo-Rizzo, P.	100	Italy	0.03
Sinonasal pain	Boscolo-Rizzo, P.	304	Italy	0.026
	Boscolo-Rizzo, P.	100	Italy	0.02
Dysphagia/swallowing difficulties	Catalán, I. P.	76	Spain	0.066
Odynophagia/painful swallowing	Catalán, I. P.	76	Spain	0.026
Wheezing	Boscolo-Rizzo, P.	304	Italy	0.02
	Boscolo-Rizzo, P.	100	Italy	0.02
Vertigo	Seeßle, J.	96	Germany	0.231
Paraesthesia	Méndez, R.	171	Spain	0.07
Edema	Liu, T.	486	China	0.002
	Zhang, X.	2433	China	0.014
Shivering	Seeßle, J.	96	Germany	0.051
Sweeting	Zhang, X.	2433	China	0.169

Symptoms	First author	Sample size	Country	Prevalence of symptom
Night sweats	Liu, T.	486	China	0.002
Tremors	Méndez, R.	171	Spain	0.012
Skin pruritus	Liu, T.	486	China	0.006
Red eyes	Boscolo-Rizzo, P.	304	Italy	0.013
	Boscolo-Rizzo, P.	100	Italy	0.01
Cold	Seeßle, J.	96	Germany	0.103
Numbness in limbs	Liu, T.	486	China	0.004
Muscle weakness	Maestre-Muñiz, M. M.	543	Spain	0.074
Hypoacusis/hearing loss	Catalán, I. P.	76	Spain	0.158
	Liu, T.	486	China	0.002
Vision loss	Liu, T.	486	China	0
	Maestrini, V.	118	Italy	0.017
Smell or taste impairment	Boscolo-Rizzo, P.	304	Italy	0.22
	Zhao, Y.	94	China	0.117
PTSD symptom	Latronico, N.	49	Italy	0.06
	Méndez, R.	171	Spain	0.246
Cognitive impairment/ neurocognitive dysfunction	Latronico, N.	45	Italy	0.16
	Méndez, R.	171	Spain	0.468

Supplementary Table S3. Sensitivity analyses for prevalence of post-COVID symptoms.

**Sensitivity analyses:**<br>**Pooled prevalence (95% CI)**	**Excluding studies rated as poor quality**	**Prevalence estimates after logit transformation**	**Prevalence estimates after Freeman-Tukey double arcsine transformation**	**Range of pooled prevalence after excluding individual studies**
Fatigue/weakness	0.28 (0.17-0.39)	0.25 (0.17-0.36)	0.27 (0.17-0.38)	0.26-0.30
Dyspnoea/breathlessness	0.19 (0.13-0.25)	0.15 (0.10-0.22)	0.17 (0.10-0.25)	0.16-0.20
Cough	0.05 (0.04-0.07)	0.06 (0.04-0.10)	0.06 (0.04-0.09)	0.05-0.07
Chest pain	0.05 (0.02-0.07)	0.05 (0.04-0.07)	0.05 (0.03-0.07)	0.04-0.05
Sore throat/difficult to swallow	-	0.03 (0.02-0.05)	0.03 (0.01-0.04)	0.02-0.03
Sputum production	0.02 (0.01-0.03)	0.02 (0.01-0.04)	0.02 (0.01-0.03)	0.02
Depression	0.20 (0.08-0.33)	0.22 (0.13-0.35)	0.22 (0.12-0.35)	0.18-0.26
Anxiety	0.19 (0.13-0.25)	0.20 (0.11-0.33)	0.21 (0.12-0.32)	0.19-0.25
Memory loss/memory complaints	0.20 (0.03-0.36)	0.13 (0.05-0.27)	0.16 (0.04-0.35)	0.14-0.24
Concentration difficulties	-	0.15 (0.03-0.47)	0.17 (0.02-0.41)	-
Insomnia/sleep difficulties	-	0.10 (0.07-0.16)	0.11 (0.06-0.17)	0.10-0.13
Loss of smell/smell disorder	0.06 (0.04-0.08)	0.06 (0.03-0.13)	0.07 (0.04-0.12)	0.05-0.09
Loss of taste/taste disorder	0.05 (0.03-0.07)	0.05 (0.02-0.10)	0.05 (0.03-0.09)	0.03-0.06
Arthromyalgia	-	0.24 (0.13-0.38)	0.24 (0.10-0.42)	-
Muscle pain	-	0.07 (0.05-0.10)	0.07 (0.04-0.11)	0.07-0.09
Joint pain	-	0.09 (0.06-0.15)	0.10 (0.05-0.16)	0.08-0.12
Back/waist pain	-	0.04 (0.02-0.07)	0.04 (0.02-0.06)	-
Headache	0.06 (0.04-0.08)	0.07 (0.04-0.12)	0.08 (0.05-0.11)	0.06-0.09
Dizziness	-	0.04 (0.03-0.06)	0.04 (0.03-0.05)	0.03-0.04
Skin rash	-	0.03 (0.02-0.05)	0.03 (0.02-0.04)	-
Hair loss	-	0.06 (0.02-0.12)	0.05 (0.01-0.14)	0.03-0.09

Palpitations	0.05 (0.03-0.08)	0.05 (0.04-0.08)	0.05 (0.03-0.08)	0.04-0.06

Note: “-” refers to not applicable.

Supplementary Figure S1. Funnel plot of meta-analysis for prevalence of post-COVID fatigue/weakness.

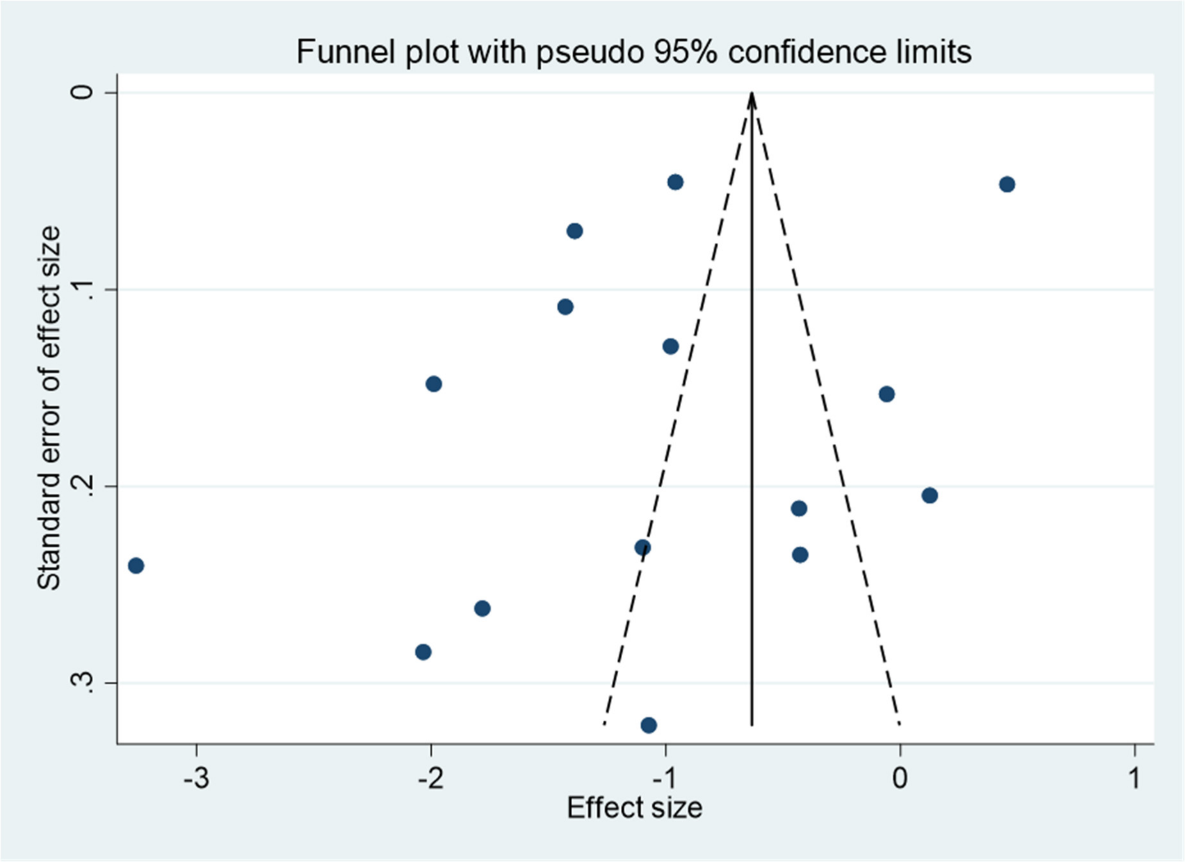

Note: The prevalence estimates were logit transformed.

Supplementary Figure S2. Funnel plot of meta-analysis for prevalence of post-COVID dyspnoea/breathlessness.

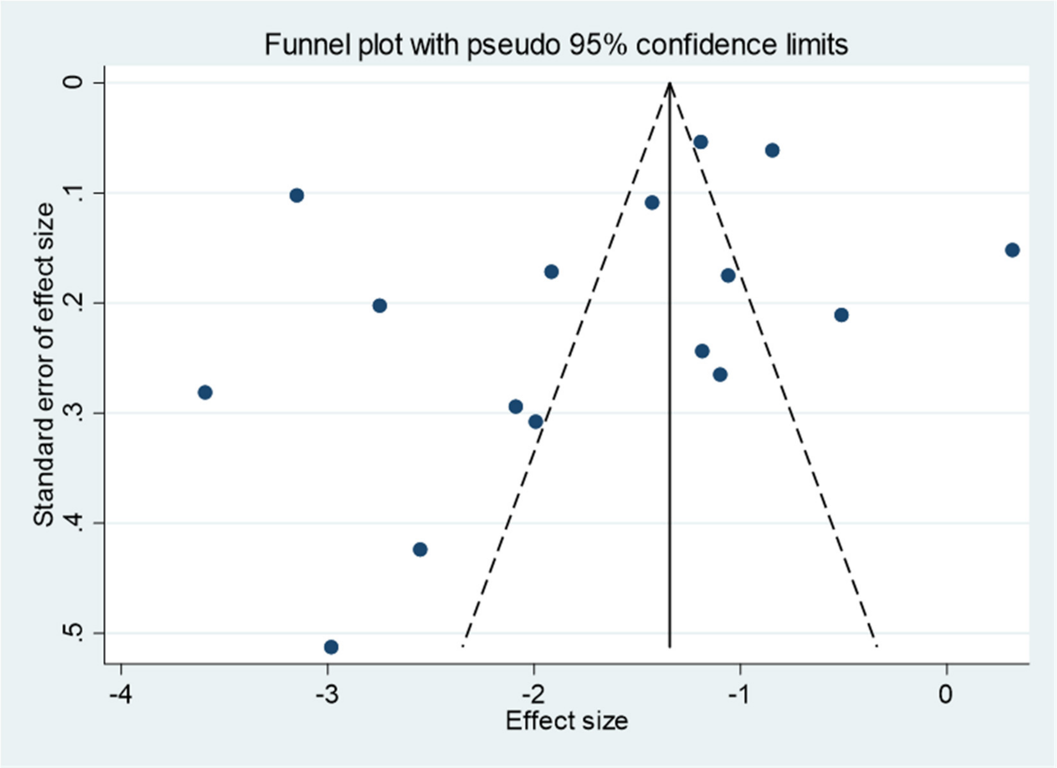

Note: The prevalence estimates were logit transformed.

Supplementary Figure S3. Funnel plot of meta-analysis for prevalence of post-COVID headache.

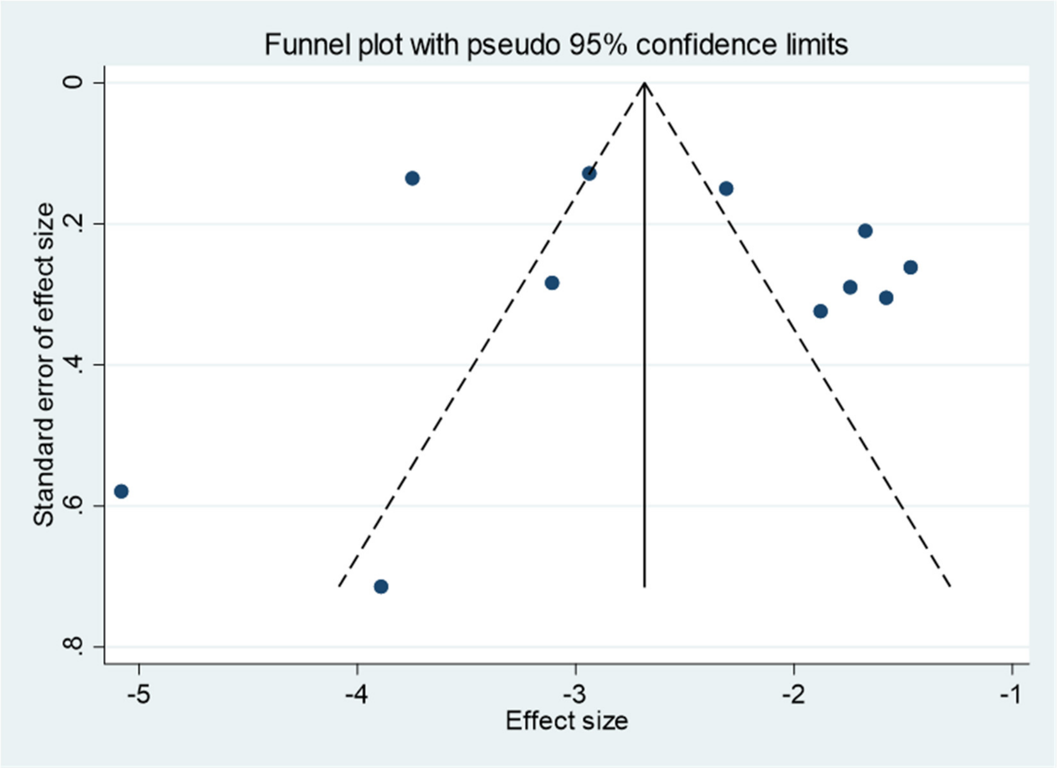

Note: The prevalence estimates were logit transformed.
